# Cecal microbiota association with tumor load in a colorectal cancer mouse model

**DOI:** 10.1080/16512235.2017.1352433

**Published:** 2017-01-01

**Authors:** Line Skute Bråten, Marianne Sødring, Jan Erik Paulsen, Lars Gustav Snipen, Knut Rudi

**Affiliations:** ^a^ Department of Chemistry, Biotechnology and Food Science, Norwegian University of Life Sciences, Ås, Norway; ^b^ Department of Food Safety and Infection Biology, Norwegian University of Life Sciences, Ås, Norway

**Keywords:** Colorectal cancer, mouse model, microbiota, 16S rRNA gene

## Abstract

**Background**: Colorectal cancer (CRC) is one of the most common cancer types worldwide. The role of the intestinal microbiota in CRC, however, is not well established. In particular, the co-variation between age, tumor progression and microbiota remains largely unknown.

**Objective and design**: We therefore used a recently developed A/J Min/+ mouse model resembling human CRC to investigate how microbial composition in cecum correlates with tumor progression, butyrate and age.

**Results**: We found that the association between the gut microbiota and tumor load was stronger, by far, than the association with both butyrate and age. The strongest direct tumor association was found for mucosal bacteria, with nearly 60% of the significantly correlating operational taxonomic units being correlated with CRC tumor load alone.

**Conclusion**: We favor a systemic association between tumor load and microbiota, since the correlations are associated with tumor load in gut segments other than the cecum (both small and large intestine).

## Introduction

Colorectal cancer (CRC) is the third most common cancer in men and the second most common cancer in women worldwide. In 2012, there were almost 1.4 million new cases and approximately 700,000 deaths reported [1]. CRC arises through a series of characterized histopathological changes in the colon, and several different signaling pathways play important roles in the development of this type of cancer [2]. The rapid renewal of the colonic epithelium also increases the risk of mutations that can lead to the development of tumors.

There are three general models for explaining the association of the gut microbiota with CRC. The alpha-bug hypothesis suggests that toxigenic bacteria induce a self-enforcing systemic response favoring the alpha-bug [3]. The driver–passenger hypothesis states that the gut microbiota association with CRC is mainly a succession process, where bacteria that are favored by the tumorigenic environment later replace the initial CRC inducers [4]. Finally, the microbiota adaptation hypothesis suggests that the association between the gut microbiota and CRC is mainly an adaptation of the gut microbiota, as a consequence of the changed environment during CRC development [5].

The mouse represents one of the most important gut microbiota models in relation to human disease [6]. Mouse models have been used to address several gut microbiota-associated diseased states, such as obesity [7] and inflammation-associated diseases [8]. Furthermore, mouse models have been used to examine the correlation between gut microbiota and aging [9], as well as the role of the gut microbiota in the host’s susceptibility to colonic tumorigenesis [10,11]. Although age, microbiota and tumorigenesis are strongly confounded, no studies have yet related age and CRC to the gut microbiota in mouse models.

The aim of this work was to investigate the co-variation between tumor progression, age, butyrate and microbiota in A/J Min/+ mice. Illumina 16S ribosomal RNA (rRNA) gene sequencing was used for the characterization of the microbiota in the cecum (lumen and mucosa), while gas chromatography (GC) was used to investigate the potential antitumor effect of butyrate.

The A/J Min/+ mouse model chosen for this study is a novel mouse model that was only recently characterized [12]. This new A/J Min/+ mouse spontaneously develops a considerable number of colonic lesions that will, with time, progress to carcinoma. The conventional C57BL/6J Min/+ mouse, on the other hand, primarily develops lesions in the small intestines, and few, if any, lesions in the colon, which seldom progress to carcinoma [13,14]. The A/J Min/+ mouse has also been shown to have the potential for at least local metastasis [12]. Taken together, these characteristics of the novel A/J Min/+ mouse more closely reflect aspects of human colorectal carcinogenesis. Other models for CRC, where tumorigenesis is induced by the addition of chemicals, also exist [15]; however, these suffer from the potential confounding effects of the chemicals. We chose to investigate the cecum microbiota, since the cecum is the main site of fermentation and microbial-derived metabolite production with the most pronounced effect on mouse health [16], particularly related to the antitumor effect of butyrate [17]. Furthermore, the tumor load in the cecum is very low, reducing the potential confounding effect of changed environment or physiology due to tumor progression (unpublished observations).

Here, we present evidence that tumor load is more important than age and butyrate in the association with the gut microbiota. From our results, we therefore favor the alpha-bug hypothesis in explaining the association between gut microbiota and CRC.

## Materials and methods

**Figure 1.**
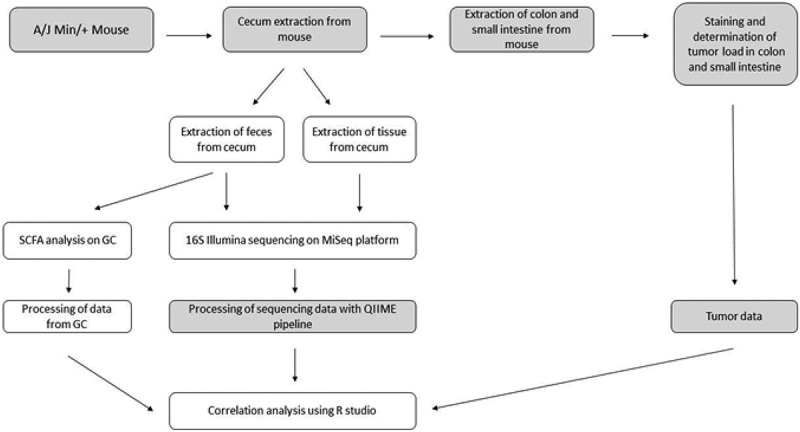
Overview illustrating the workflow of this project. Tasks in the gray areas were performed before the start of the project or by an external part of the project. SCFA, short-chain fatty acid; GC, gas chromatography.

An outline of samples and analyses is given in Figure 1. This study was conducted in strict accordance with the Norwegian Regulation on Animal Experimentation, and approved by the Institutional Animal Care and Use Committee at the Norwegian University of Life Sciences, Campus Adamstuen.

### Mouse samples

For this study we used a Min/+ mouse strain that is well suited as a model for human CRC [12]. The mouse strain, called A/J Min/+, was established at the Norwegian Institute of Public Health, and is the result of a backcross with C57BL6/J Min/+ mice and A/J wild-type mice for more than 12 generations. The A/J Min/+ mouse has been maintained at the Norwegian University of Life Sciences (Adamstuen, Norway) as an inbred colony for several years [12,18]. Cecum from 68 mice from a previous study [12] and an ongoing study at the same institute was used in this project. The housing facilities and experimental conditions were similar across the studies. Samples were preserved in STAR buffer (Roche Diagnostics, Basel, Switzerland) according to the producer’s recommendations before further processing.

### Sample processing

Lysis and DNA extraction was performed as previously described using the mag™ midi kit (LGC Genomics, Berlin, Germany) [19]. The V3 and V4 region of the 16S rRNA gene was amplified using forward primer PRK341 and reverse primer PRK806 [20], with further processing for Illumina sequencing as previously described [19]. Finally, quantification of butyric acid in cecum content was performed by GC [21]. Butyric acid was chosen as a biomarker because this is the most well-documented short-chain fatty acid to be protective against CRC [17].

### Data analysis

The Illumina sequencing data were analyzed using a standard workflow from a Quantitative Insights Into Microbial Ecology (QIIME) pipeline [22]. In the first step, paired-end reads were combined and clustered with 97% identity level using *usearch* v. 7 [23], which implements the error-minimizing *uparse* algorithm [24] against the Greengenes v. 13.8 database [25].

Statistical analyses were performed using RStudio (https://www.rstudio.com/), with R v. 3.2.2 (https://www.r-project.org/) and package Vegan v. 2.3-0 (cran.r-project.org/web/packages/vegan/). All statistical tests were conducted at a 95% confidence level after correction for multiple testing by controlling the false discovery rate (FDR).

## Results

### Library characteristics, taxonomic composition and diversity

In total, 2,495,371 sequences were obtained from all samples (*n* = 188); on average, this corresponded to 13,273 sequences per sample with a standard deviation of 10,742. The operational taxonomic unit (OTU) table was rarified at 2000 sequences per sample. The library contained a total of 326 OTUs distributed in seven phyla and a total of 13 bacterial classes (Supplementary Table 1).

**Figure 2.**
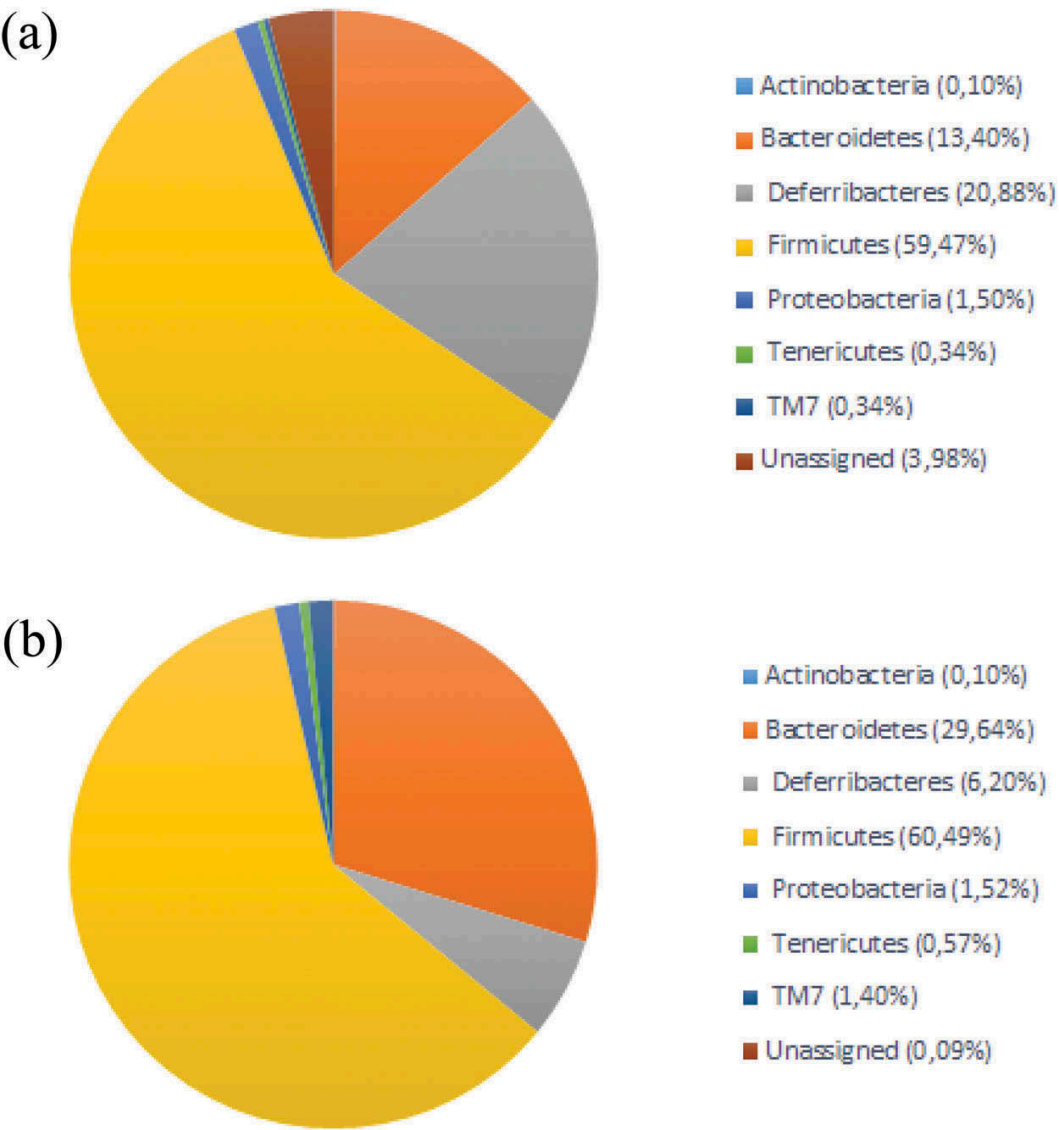
Taxonomic composition at phylum level in (a) cecum mucosa and (b) cecum content.

**Figure 3.**
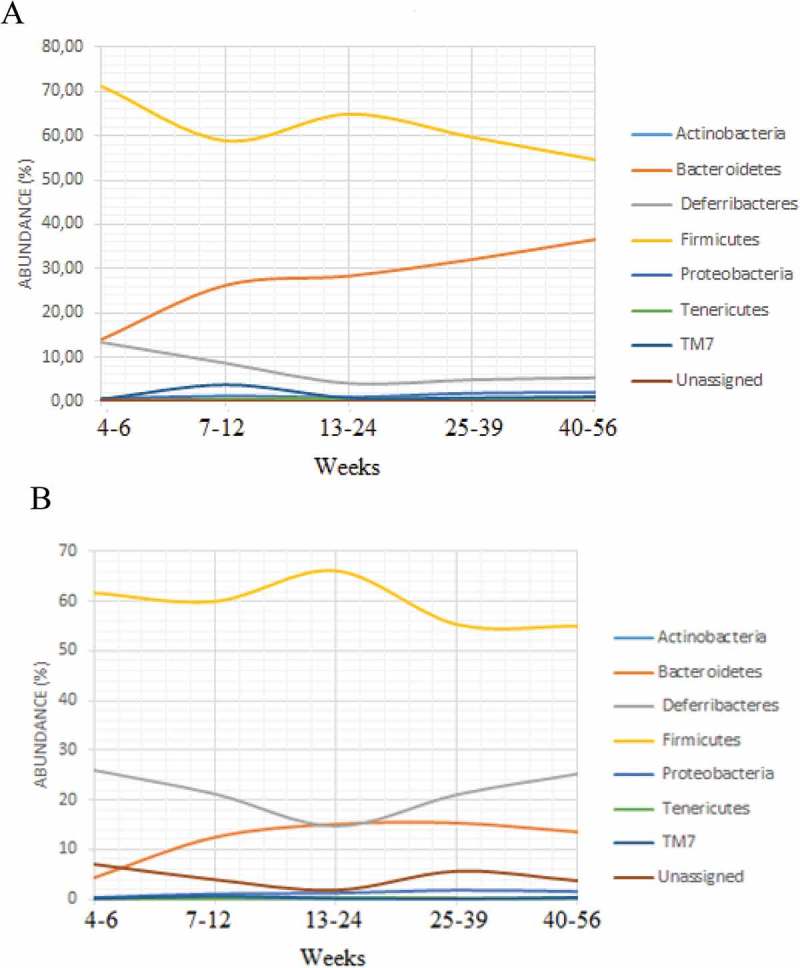
Relative abundance of each phylum and how the abundance varies with age in (**a**) cecum content and (b) tissue samples.

**Figure 4.**
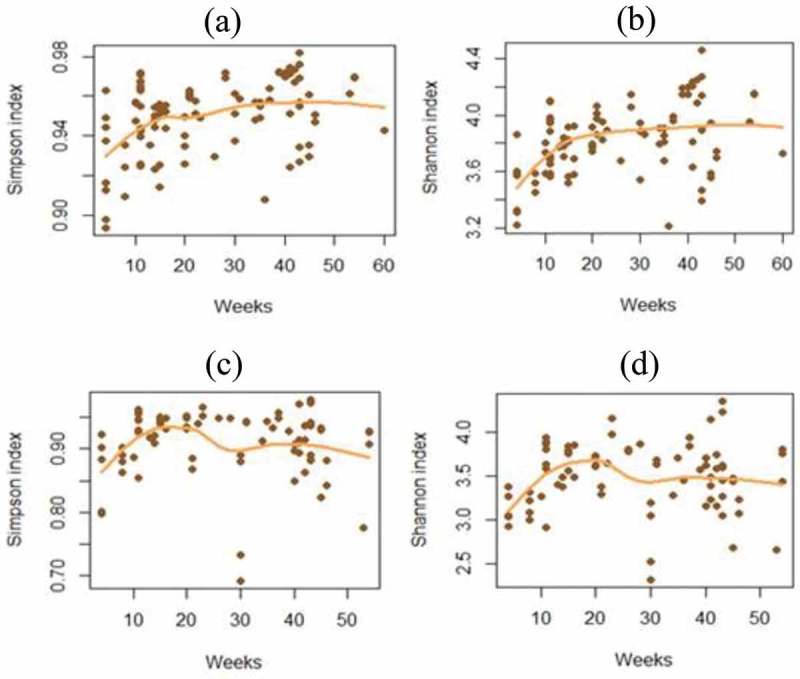
Calculated alpha-diversity using the Shannon and Simpson indices. (a) Simpson index in cecum content; (b) Shannon index in cecum content; (c) Simpson index in tissue samples; (d) Shannon index in tissue samples.

Firmicutes was the most dominant phylum in both cecum content and mucosa. Bacteroidetes was the second most dominant phylum in cecum content, while in the mucosa, this was found to be bacteria belonging to the phylum Deferribacteres (Figure 2). For the mucosal samples, there were no major changes in the microbiota composition with age, while for the lumen samples there was an increase in Bacteroidetes and a decrease in Firmicutes (Figure 3). For the alpha-diversity, there were increases in diversity up to 20 weeks (Figure 4) for both mucosa and cecum, while the beta-diversity did not show any clear clustering patterns (results not shown).

### Correlations between microbiota, tumor load and age

**Figure 5.**
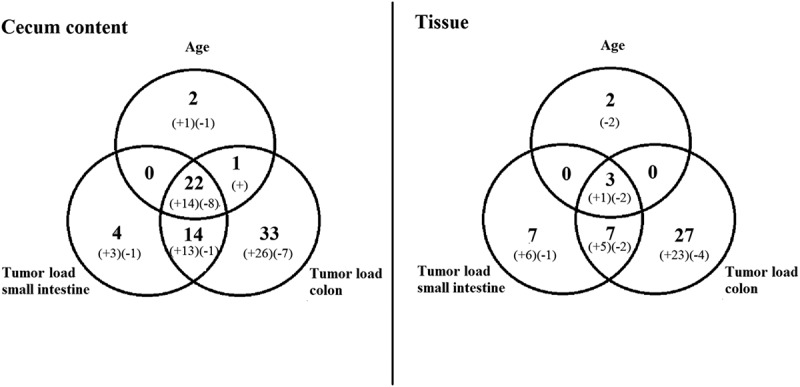
Number of operational taxonomic units (OTUs) with significant correlation with age, tumor load in colon and tumor load in small intestine. Left: samples from cecum content; right: tissue samples. Numbers in parentheses indicate whether the correlations are positive (+) or negative (–).

In total, 76 OTUs were significantly correlated with age and/or tumor progression (small and large intestine) in samples from cecum content, and 66 OTUs for mucosa samples (*p* < 0.05, FDR-corrected Spearman correlation) (Figure 5). In the cecum content, 20 OTUs belonging to the S24-7 family (phylum Bacteroidetes) were positively correlated either with age and/or with tumor progression in both small intestine and colon. Similarly, 12 of the positively correlating OTUs belonged to S24-7 for the mucosal samples. We were not able to extract systematic information for other phylogroups. All significant FDR-corrected correlations are shown in Supplementary Table 2.

### Correlations between microbiota, tumor load and butyrate

The level of butyrate was 13.1 ± 7 mM (mean ± SD) for the cecum content, while there were no significant correlations between butyrate levels and tumor load. However, two OTUs were negatively correlated with butyrate: OTU 69 was classified as Ruminococcaceae (*p* = 0.003, FDR-corrected Spearman correlation) in cecum content, and OTU 220 was classified as Ruminococcus (*p* = 0.045, FDR-corrected Spearman correlation) in tissue samples.

## Discussion

There were major associations between gut microbiota and tumor load, seemingly overruling the associations with both age [12] and butyrate [17]. OTUs belonging to the Bacteroidetes family S24-7 showed the majority of the positive correlations with tumor load. An overrepresentation of Bacteroidetes has previously been reported in several studies related to human CRC [26,27], as well as in small intestinal tumors in adenomatous polyposis coli (APC) Min/+ mice [28]. With regard to potential mechanisms, Bacteroidetes have been suggested to be strains that can trigger CRC through systemic modulations [29]. The main CRC-correlating Bacteriodetes family S24-7 has previously been associated with liver injury and TLR4 signaling, which points towards a role in inflammation [30]. Therefore, inflammation can be a potential trigger of CRC in the A/J Min/+ mouse model [31].

Regarding the association between microbiota and CRC, the alpha-bug hypothesis [3] is the most likely explanation. Most of the mucosa-associated strains showed a correlation with tumor load in both large and small intestine. Therefore, it is unlikely that the association is a consequence of adaptation to the changed environment due to the tumorigenesis (microbiota adaptation hypothesis) or a succession process (driver–passenger hypothesis). One hypothesis is that the association is due to the triggering of systemic tumorigenic conditions by key members of the microbiota, as in the concept of the alpha-bug hypothesis, potentially through the induction of inflammation [30].

Although mouse models have been used to investigate a range of gut microbiota-associated diseases, the mouse gut microbiota still remains poorly characterized [6]. In this study, Deferribacteres was found to be the second-most dominant phylum in the mucosal samples. This phylum is most likely to be represented by *Mucispirillum schaedleri*, which has previously been associated with mucosa and potential translocation from the intestine to the hepatobiliary system in laboratory mice [32]. However, very little is known about its role in health and disease.

In conclusion, our work provides evidence for a strong correlation between gut bacteria and tumor load in a novel CRC model, thereby supporting the alpha-bug hypothesis. Thus, the A/J Min/+ mouse CRC model could form the basis for general mechanistic insights into how the microbiota is associated with CRC development.

## Supplementary Material

Supplementary material

## References

[1] Ferlay J, Steliarova-Foucher E, Lortet-Tieulent J, et al Cancer incidence and mortality patterns in Europe: estimates for 40 countries in 2012. Eur J Cancer. 2013 4;49(6):1374–1403. PubMed PMID: 23485231. 23485231 10.1016/j.ejca.2012.12.027

[2] Roy S, Majumdar AP. Signaling in colon cancer stem cells. J Mol Signal. 2012;7(1):11 PubMed PMID: 22866952; PubMed Central PMCID: PMCPMC3485105. 22866952 10.1186/1750-2187-7-11 PMC3485105

[3] Sears CL, Pardoll DM Perspective: alpha-bugs, their microbial partners, and the link to colon cancer. J Infect Dis. 2011 2 1;203(3):306–311. PubMed PMID: 21208921; PubMed Central PMCID: PMCPMC3071114. 21208921 10.1093/jinfdis/jiq061 PMC3071114

[4] Tjalsma H, Boleij A, Marchesi JR, et al A bacterial driver-passenger model for colorectal cancer: beyond the usual suspects. Nat Reviews. 2012 8;10(8):575–582. PubMed PMID: 22728587. 10.1038/nrmicro2819 22728587

[5] Yu YN, Fang JY Gut microbiota and colorectal cancer. Gastrointest Tumors. 2015 5;2(1):26–32. PubMed PMID: 26674881; PubMed Central PMCID: PMCPMC4668798. 26674881 10.1159/000380892 PMC4668798

[6] Xiao L, Feng Q, Liang S, et al A catalog of the mouse gut metagenome. Nat Biotechnol. 2015 10;33(10):1103–1108. PubMed PMID: 26414350. 26414350 10.1038/nbt.3353

[7] Walker A, Pfitzner B, Neschen S, et al Distinct signatures of host-microbial meta-metabolome and gut microbiome in two C57BL/6 strains under high-fat diet. ISME J. 2014 12;8(12):2380–2396. PubMed PMID: 24906017; PubMed Central PMCID: PMCPMC4260703. 24906017 10.1038/ismej.2014.79 PMC4260703

[8] Gkouskou KK, Deligianni C, Tsatsanis C, et al The gut microbiota in mouse models of inflammatory bowel disease. Front Cell Infect Microbiol. 2014;4:28 PubMed Central PMCID: PMCPMC3937555. 24616886 10.3389/fcimb.2014.00028 PMC3937555

[9] Langille MG, Meehan CJ, Koenig JE, et al Microbial shifts in the aging mouse gut. Microbiome. 2014;2(1):50 PubMed PMID: 25520805; PubMed Central PMCID: PMCPMC4269096. 25520805 10.1186/s40168-014-0050-9 PMC4269096

[10] Baxter NT, Zackular JP, Chen GY, et al Structure of the gut microbiome following colonization with human feces determines colonic tumor burden. Microbiome. 2014;2:20 PubMed PMID: 24967088; PubMed Central PMCID: PMCPMC4070349. 24967088 10.1186/2049-2618-2-20 PMC4070349

[11] Moen B, Henjum K, Mage I, et al Effect of dietary fibers on cecal microbiota and intestinal tumorigenesis in azoxymethane treated A/J Min/+ Mice. PLoS ONE. 2016;11(5):e0155402 PubMed PMID: 27196124. 27196124 10.1371/journal.pone.0155402 PMC4873001

[12] Sodring M, Gunnes G, Paulsen JE Spontaneous initiation, promotion and progression of colorectal cancer in the novel A/J Min/+ mouse. Int J Cancer. 2016 4 15;138(8):1936–1946. PubMed PMID: 26566853. 26566853 10.1002/ijc.29928

[13] Moser AR, Pitot HC, Dove WF A dominant mutation that predisposes to multiple intestinal neoplasia in the mouse. Science (New York, NY. 1990 1 19;247(4940):322–324. PubMed PMID: 2296722. 10.1126/science.2296722 2296722

[14] Halberg RB, Waggoner J, Rasmussen K, et al Long-lived Min mice develop advanced intestinal cancers through a genetically conservative pathway. Cancer Res. 2009 7 15;69(14):5768–5775. PubMed PMID: 19584276; PubMed Central PMCID: PMCPMC2775466. 19584276 10.1158/0008-5472.CAN-09-0446 PMC2775466

[15] Tanaka T, Kohno H, Suzuki R, et al A novel inflammation-related mouse colon carcinogenesis model induced by azoxymethane and dextran sodium sulfate. Cancer Sci. 2003 11;94(11):965–973. PubMed PMID: 14611673. 14611673 10.1111/j.1349-7006.2003.tb01386.x PMC11160237

[16] Nguyen TL, Vieira-Silva S, Liston A, et al How informative is the mouse for human gut microbiota research? Dis Model Mech. 2015 1;8(1):1–16. PubMed PMID: 25561744; PubMed Central PMCID: PMCPMC4283646. 25561744 10.1242/dmm.017400 PMC4283646

[17] Louis P, Hold GL, Flint HJ The gut microbiota, bacterial metabolites and colorectal cancer. Nat Reviews. 2014 10;12(10):661–672. PubMed PMID: 25198138. 10.1038/nrmicro3344 25198138

[18] Sodring M, Oostindjer M, Egelandsdal B, et al Effects of hemin and nitrite on intestinal tumorigenesis in the A/J Min/+ mouse model. PLoS ONE. 2015;10(4):e0122880 PubMed PMID: 25836260; PubMed Central PMCID: PMCPMC4383626. 25836260 10.1371/journal.pone.0122880 PMC4383626

[19] Avershina E, Lundgard K, Sekelja M, et al Transition from infant- to adult-like gut microbiota. Environmen Microbiol. 2016;18(7):2226–2236. 10.1111/1462-2920.13248 26913851

[20] Yu Y, Lee C, Kim J, et al Group-specific primer and probe sets to detect methanogenic communities using quantitative real-time polymerase chain reaction. Biotechnol Bioeng. 2005 3 20;89(6):670–679. PubMed PMID: ISI:000227247700006. 15696537 10.1002/bit.20347

[21] Szczesniak O, Hestad KA, Hanssen JF, et al Isovaleric acid in stool correlates with human depression. Nutrit Neurosci. 2016;19(7):279–283. 10.1179/1476830515Y.0000000007 25710209

[22] Caporaso JG, Kuczynski J, Stombaugh J, et al QIIME allows analysis of high-throughput community sequencing data. Nat Methods. 2010 5;7(5):335–336. PubMed PMID: 20383131; PubMed Central PMCID: PMC3156573. 20383131 10.1038/nmeth.f.303 PMC3156573

[23] Edgar RC Search and clustering orders of magnitude faster than BLAST. Bioinformatics. 2010 10 1;26(19):2460–2461. PubMed PMID: 20709691. 20709691 10.1093/bioinformatics/btq461

[24] Edgar RC UPARSE: highly accurate OTU sequences from microbial amplicon reads. Nat Methods. 2013 10;10(10):996–998. PubMed PMID: 23955772. 23955772 10.1038/nmeth.2604

[25] DeSantis TZ, Hugenholtz P, Larsen N, et al Greengenes, a chimera-checked 16S rRNA gene database and workbench compatible with ARB. Appl Environ Microbiol. 2006 7;72(7):5069–5072. PubMed PMID: 16820507; PubMed Central PMCID: PMC1489311. 16820507 10.1128/AEM.03006-05 PMC1489311

[26] Marchesi JR, Dutilh BE, Hall N, et al Towards the human colorectal cancer microbiome. PLoS ONE. 2011;6(5):e20447 PubMed PMID: 21647227; PubMed Central PMCID: PMCPMC3101260. 21647227 10.1371/journal.pone.0020447 PMC3101260

[27] Sobhani I, Tap J, Roudot-Thoraval F, et al Microbial dysbiosis in colorectal cancer (CRC) patients. PLoS ONE. 2011;6(1):e16393 PubMed PMID: 21297998; PubMed Central PMCID: PMCPMC3029306. 21297998 10.1371/journal.pone.0016393 PMC3029306

[28] Son JS, Khair S, Pettet DW 3rd, et al. Altered interactions between the gut microbiome and colonic mucosa precede polyposis in APCMin/+ Mice. PLoS ONE. 2015;10(6):e0127985 PubMed PMID: 26121046; PubMed Central PMCID: PMCPMC4485894. 26121046 10.1371/journal.pone.0127985 PMC4485894

[29] Yu H, Kortylewski M, Pardoll D Crosstalk between cancer and immune cells: role of STAT3 in the tumour microenvironment. Nat Rev Immunol. 2007 1;7(1):41–51. PubMed PMID: 17186030. 17186030 10.1038/nri1995

[30] Harris JK, El Kasmi KC, Anderson AL, et al Specific microbiome changes in a mouse model of parenteral nutrition associated liver injury and intestinal inflammation. PLoS ONE. 2014;9(10):e110396 PubMed PMID: 25329595; PubMed Central PMCID: PMCPMC4203793. 25329595 10.1371/journal.pone.0110396 PMC4203793

[31] Li YH, Kundu P, Seow SW, et al Gut microbiota accelerate tumor growth via c-jun and STAT3 phosphorylation in APC(Min/+) mice. Carcinogenesis. 2012 11;33(6):1231–1238. PubMed PMID: WOS:000306135800015. 22461519 10.1093/carcin/bgs137

[32] Robertson BR, O’Rourke JL, Neilan BA, et al Mucispirillum schaedleri gen. nov., sp. nov., a spiral-shaped bacterium colonizing the mucus layer of the gastrointestinal tract of laboratory rodents. Int J Syst Evol Microbiol. 2005 5;55(Pt 3):1199–1204. PubMed PMID: 15879255. 15879255 10.1099/ijs.0.63472-0

