# Supplementary material for: Cecal microbiota association with tumor load in a colorectal cancer mouse model
Source: Microb Ecol Health Dis. 2017 Jan 1;28(1):1352433. doi: 10.1080/16512235.2017.1352433 (PMC5614384; doi:10.1080/16512235.2017.1352433)
Supplement: Supplementary material [file ZMEH_A_1352433_SM1031.docx]

**SUPPLEMENTARY TABLES**

**Supplementary Table 1.** All OTUs detected in 16S rRNA analysis with assigned taxonomy.

| **Phylum** | **Class** | **Order** | **Family** | **Genus** | **Species** | **OTU** |
| --- | --- | --- | --- | --- | --- | --- |
| Actinobacteria | Coriobacteriia | Coriobacteriales | Coriobacteriaceae | Adlercreutzia |  | 217 |
| Actinobacteria | Coriobacteriia | Coriobacteriales | Coriobacteriaceae | Adlercreutzia |  | 243 |
| Actinobacteria | Coriobacteriia | Coriobacteriales | Coriobacteriaceae | Adlercreutzia |  | 251 |
| Actinobacteria | Coriobacteriia | Coriobacteriales | Coriobacteriaceae | Adlercreutzia |  | 252 |
| Actinobacteria | Coriobacteriia | Coriobacteriales | Coriobacteriaceae | Adlercreutzia |  | 256 |
| Actinobacteria | Coriobacteriia | Coriobacteriales | Coriobacteriaceae | Adlercreutzia |  | 270 |
| Actinobacteria | Actinobacteria | Bifidobacteriales | Bifidobacteriaceae | Bifidobacterium | pseudolongum | 292 |
| Actinobacteria | Coriobacteriia | Coriobacteriales | Coriobacteriaceae | Adlercreutzia |  | 301 |
| Actinobacteria | Coriobacteriia | Coriobacteriales | Coriobacteriaceae | Adlercreutzia |  | 310 |
| Actinobacteria | Coriobacteriia | Coriobacteriales | Coriobacteriaceae | Adlercreutzia |  | 317 |
| Bacteroidetes | Bacteroidia | Bacteroidales | Bacteroidaceae | Bacteroides |  | 6 |
| Bacteroidetes | Bacteroidia | Bacteroidales | Rikenellaceae |  |  | 7 |
| Bacteroidetes | Bacteroidia | Bacteroidales | S24-7 |  |  | 9 |
| Bacteroidetes | Bacteroidia | Bacteroidales | S24-7 |  |  | 12 |
| Bacteroidetes | Bacteroidia | Bacteroidales | Rikenellaceae |  |  | 13 |
| Bacteroidetes | Bacteroidia | Bacteroidales | S24-7 |  |  | 17 |
| Bacteroidetes | Bacteroidia | Bacteroidales | Rikenellaceae |  |  | 19 |
| Bacteroidetes | Bacteroidia | Bacteroidales | Bacteroidaceae | Bacteroides |  | 22 |
| Bacteroidetes | Bacteroidia | Bacteroidales | S24-7 |  |  | 29 |
| Bacteroidetes | Bacteroidia | Bacteroidales | S24-7 |  |  | 30 |
| Bacteroidetes | Bacteroidia | Bacteroidales | S24-7 |  |  | 41 |
| Bacteroidetes | Bacteroidia | Bacteroidales | S24-7 |  |  | 47 |
| Bacteroidetes | Bacteroidia | Bacteroidales | S24-7 |  |  | 49 |
| Bacteroidetes | Bacteroidia | Bacteroidales | S24-7 |  |  | 51 |
| Bacteroidetes | Bacteroidia | Bacteroidales | Rikenellaceae |  |  | 52 |
| Bacteroidetes | Bacteroidia | Bacteroidales | Rikenellaceae | AF12 |  | 57 |
| Bacteroidetes | Bacteroidia | Bacteroidales | [Paraprevotellaceae] | [Prevotella] |  | 59 |
| Bacteroidetes | Bacteroidia | Bacteroidales | Porphyromonadaceae | Parabacteroides | distasonis | 64 |
| Bacteroidetes | Bacteroidia | Bacteroidales | Rikenellaceae |  |  | 65 |
| Bacteroidetes | Bacteroidia | Bacteroidales | [Odoribacteraceae] | Odoribacter |  | 75 |
| Bacteroidetes | Bacteroidia | Bacteroidales |  |  |  | 77 |
| Bacteroidetes | Bacteroidia | Bacteroidales | S24-7 |  |  | 80 |
| Bacteroidetes | Bacteroidia | Bacteroidales | S24-7 |  |  | 82 |
| Bacteroidetes | Bacteroidia | Bacteroidales | Bacteroidaceae | Bacteroides | acidifaciens | 87 |
| Bacteroidetes | Bacteroidia | Bacteroidales | S24-7 |  |  | 88 |
| Bacteroidetes | Bacteroidia | Bacteroidales | S24-7 |  |  | 90 |
| Bacteroidetes | Bacteroidia | Bacteroidales | S24-7 |  |  | 94 |

| Bacteroidetes | Bacteroidia | Bacteroidales | S24-7 |  |  | 98 |
| --- | --- | --- | --- | --- | --- | --- |
| Bacteroidetes | Bacteroidia | Bacteroidales | S24-7 |  |  | 100 |
| Bacteroidetes | Bacteroidia | Bacteroidales | S24-7 |  |  | 105 |
| Bacteroidetes | Bacteroidia | Bacteroidales | S24-7 |  |  | 107 |
| Bacteroidetes | Bacteroidia | Bacteroidales | Rikenellaceae |  |  | 110 |
| Bacteroidetes | Bacteroidia | Bacteroidales | Rikenellaceae |  |  | 119 |
| Bacteroidetes | Bacteroidia | Bacteroidales | S24-7 |  |  | 121 |
| Bacteroidetes | Bacteroidia | Bacteroidales | Bacteroidaceae | Bacteroides |  | 123 |
| Bacteroidetes | Bacteroidia | Bacteroidales | S24-7 |  |  | 124 |
| Bacteroidetes | Bacteroidia | Bacteroidales | Porphyromonadaceae | Parabacteroides |  | 125 |
| Bacteroidetes | Bacteroidia | Bacteroidales | S24-7 |  |  | 127 |
| Bacteroidetes | Bacteroidia | Bacteroidales | S24-7 |  |  | 140 |
| Bacteroidetes | Bacteroidia | Bacteroidales | S24-7 |  |  | 148 |
| Bacteroidetes | Bacteroidia | Bacteroidales | S24-7 |  |  | 151 |
| Bacteroidetes | Bacteroidia | Bacteroidales | S24-7 |  |  | 154 |
| Bacteroidetes | Bacteroidia | Bacteroidales | S24-7 |  |  | 157 |
| Bacteroidetes | Bacteroidia | Bacteroidales | S24-7 |  |  | 164 |
| Bacteroidetes | Bacteroidia | Bacteroidales | Rikenellaceae |  |  | 173 |
| Bacteroidetes | Bacteroidia | Bacteroidales | Bacteroidaceae | Bacteroides |  | 175 |
| Bacteroidetes | Bacteroidia | Bacteroidales | S24-7 |  |  | 193 |
| Bacteroidetes | Bacteroidia | Bacteroidales | S24-7 |  |  | 194 |
| Bacteroidetes | Bacteroidia | Bacteroidales | Prevotellaceae | Prevotella |  | 206 |
| Bacteroidetes | Bacteroidia | Bacteroidales | S24-7 |  |  | 207 |
| Bacteroidetes | Bacteroidia | Bacteroidales |  |  |  | 211 |
| Bacteroidetes | Bacteroidia | Bacteroidales | S24-7 |  |  | 226 |
| Bacteroidetes | Bacteroidia | Bacteroidales | S24-7 |  |  | 228 |
| Bacteroidetes | Bacteroidia | Bacteroidales | Porphyromonadaceae | Parabacteroides |  | 229 |
| Bacteroidetes | Bacteroidia | Bacteroidales | Rikenellaceae |  |  | 250 |
| Bacteroidetes | Bacteroidia | Bacteroidales |  |  |  | 263 |
| Bacteroidetes | Bacteroidia | Bacteroidales | S24-7 |  |  | 268 |
| Bacteroidetes | Bacteroidia | Bacteroidales | S24-7 |  |  | 275 |
| Bacteroidetes | Bacteroidia | Bacteroidales | Porphyromonadaceae | Parabacteroides | distasonis | 285 |
| Bacteroidetes | Bacteroidia | Bacteroidales | [Odoribacteraceae] | Odoribacter |  | 307 |
| Deferribacteres | Deferribacteres | Deferribacterales | Deferribacteraceae | Mucispirillum | schaedleri | 1 |
| Deferribacteres | Deferribacteres | Deferribacterales | Deferribacteraceae | Mucispirillum | schaedleri | 199 |
| Firmicutes | Clostridia | Clostridiales |  |  |  | 3 |
| Firmicutes | Clostridia | Clostridiales |  |  |  | 4 |
| Firmicutes | Clostridia | Clostridiales |  |  |  | 8 |
| Firmicutes | Clostridia | Clostridiales | Lachnospiraceae |  |  | 10 |
| Firmicutes | Clostridia | Clostridiales |  |  |  | 11 |
| Firmicutes | Bacilli | Lactobacillales | Lactobacillaceae | Lactobacillus |  | 14 |
| Firmicutes | Clostridia | Clostridiales | Lachnospiraceae |  |  | 15 |
| Firmicutes | Clostridia | Clostridiales |  |  |  | 16 |

| Firmicutes | Clostridia | Clostridiales | Lachnospiraceae |  |  | 18 |
| --- | --- | --- | --- | --- | --- | --- |
| Firmicutes | Clostridia | Clostridiales | Lachnospiraceae |  |  | 20 |
| Firmicutes | Clostridia | Clostridiales |  |  |  | 21 |
| Firmicutes | Clostridia | Clostridiales | Lachnospiraceae |  |  | 24 |
| Firmicutes | Clostridia | Clostridiales | Lachnospiraceae |  |  | 25 |
| Firmicutes | Clostridia | Clostridiales | Ruminococcaceae | Oscillospira |  | 26 |
| Firmicutes | Clostridia | Clostridiales |  |  |  | 27 |
| Firmicutes | Clostridia | Clostridiales | Lachnospiraceae | [Ruminococcus] | gnavus | 28 |
| Firmicutes | Clostridia | Clostridiales |  |  |  | 31 |
| Firmicutes | Clostridia | Clostridiales |  |  |  | 32 |
| Firmicutes | Clostridia | Clostridiales | Ruminococcaceae |  |  | 33 |
| Firmicutes | Clostridia | Clostridiales |  |  |  | 34 |
| Firmicutes | Clostridia | Clostridiales |  |  |  | 35 |
| Firmicutes | Clostridia | Clostridiales | Lachnospiraceae |  |  | 36 |
| Firmicutes | Clostridia | Clostridiales |  |  |  | 37 |
| Firmicutes | Clostridia | Clostridiales |  |  |  | 39 |
| Firmicutes | Clostridia | Clostridiales | Lachnospiraceae |  |  | 40 |
| Firmicutes | Clostridia | Clostridiales |  |  |  | 42 |
| Firmicutes | Clostridia | Clostridiales | Ruminococcaceae | Oscillospira |  | 43 |
| Firmicutes | Clostridia | Clostridiales |  |  |  | 46 |
| Firmicutes | Clostridia | Clostridiales | Clostridiaceae | Candidatus Arthromitus |  | 48 |
| Firmicutes | Clostridia | Clostridiales | Ruminococcaceae |  |  | 50 |
| Firmicutes | Clostridia | Clostridiales |  |  |  | 53 |
| Firmicutes | Clostridia | Clostridiales | Ruminococcaceae |  |  | 54 |
| Firmicutes | Clostridia | Clostridiales | Ruminococcaceae | Ruminococcus |  | 55 |
| Firmicutes | Clostridia | Clostridiales |  |  |  | 56 |
| Firmicutes | Clostridia | Clostridiales | Lachnospiraceae |  |  | 60 |
| Firmicutes | Clostridia | Clostridiales |  |  |  | 61 |
| Firmicutes | Clostridia | Clostridiales | Lachnospiraceae |  |  | 62 |
| Firmicutes | Clostridia | Clostridiales | Ruminococcaceae | Ruminococcus |  | 66 |
| Firmicutes | Clostridia | Clostridiales | Lachnospiraceae |  |  | 67 |
| Firmicutes | Clostridia | Clostridiales |  |  |  | 68 |
| Firmicutes | Clostridia | Clostridiales | Ruminococcaceae |  |  | 69 |
| Firmicutes | Clostridia | Clostridiales |  |  |  | 70 |
| Firmicutes | Clostridia | Clostridiales | Ruminococcaceae | Oscillospira |  | 71 |
| Firmicutes | Bacilli | Lactobacillales | Lactobacillaceae | Lactobacillus |  | 72 |
| Firmicutes | Clostridia | Clostridiales |  |  |  | 74 |
| Firmicutes | Clostridia | Clostridiales |  |  |  | 76 |
| Firmicutes | Clostridia | Clostridiales | Ruminococcaceae | Oscillospira |  | 78 |
| Firmicutes | Clostridia | Clostridiales | Lachnospiraceae |  |  | 79 |
| Firmicutes | Erysipelotrichi | Erysipelotrichales | Erysipelotrichaceae |  |  | 83 |
| Firmicutes | Clostridia | Clostridiales |  |  |  | 84 |
| Firmicutes | Clostridia | Clostridiales |  |  |  | 85 |

| Firmicutes | Clostridia | Clostridiales | Ruminococcaceae | Oscillospira |  | 86 |
| --- | --- | --- | --- | --- | --- | --- |
| Firmicutes | Clostridia | Clostridiales | Lachnospiraceae |  |  | 89 |
| Firmicutes | Clostridia | Clostridiales | Lachnospiraceae | Coprococcus |  | 91 |
| Firmicutes | Clostridia | Clostridiales |  |  |  | 92 |
| Firmicutes | Clostridia | Clostridiales |  |  |  | 93 |
| Firmicutes | Clostridia | Clostridiales | Lachnospiraceae |  |  | 95 |
| Firmicutes | Clostridia | Clostridiales |  |  |  | 96 |
| Firmicutes | Clostridia | Clostridiales |  |  |  | 97 |
| Firmicutes | Clostridia | Clostridiales |  |  |  | 99 |
| Firmicutes | Clostridia | Clostridiales |  |  |  | 101 |
| Firmicutes | Clostridia | Clostridiales |  |  |  | 102 |
| Firmicutes | Clostridia | Clostridiales |  |  |  | 103 |
| Firmicutes | Clostridia | Clostridiales |  |  |  | 104 |
| Firmicutes | Clostridia | Clostridiales | Lachnospiraceae |  |  | 106 |
| Firmicutes | Clostridia | Clostridiales |  |  |  | 108 |
| Firmicutes | Clostridia | Clostridiales | Lachnospiraceae |  |  | 109 |
| Firmicutes | Clostridia | Clostridiales | Ruminococcaceae | Ruminococcus |  | 111 |
| Firmicutes | Clostridia | Clostridiales |  |  |  | 112 |
| Firmicutes | Clostridia | Clostridiales | Ruminococcaceae | Oscillospira |  | 113 |
| Firmicutes | Clostridia | Clostridiales |  |  |  | 114 |
| Firmicutes | Bacilli | Lactobacillales | Lactobacillaceae | Lactobacillus | reuteri | 115 |
| Firmicutes | Clostridia | Clostridiales | Lachnospiraceae | [Ruminococcus] | gnavus | 116 |
| Firmicutes | Clostridia | Clostridiales |  |  |  | 117 |
| Firmicutes | Clostridia | Clostridiales |  |  |  | 118 |
| Firmicutes | Clostridia | Clostridiales | Ruminococcaceae | Ruminococcus |  | 120 |
| Firmicutes | Clostridia | Clostridiales |  |  |  | 122 |
| Firmicutes | Clostridia | Clostridiales |  |  |  | 126 |
| Firmicutes | Clostridia | Clostridiales |  |  |  | 128 |
| Firmicutes | Clostridia | Clostridiales |  |  |  | 129 |
| Firmicutes | Clostridia | Clostridiales |  |  |  | 130 |
| Firmicutes | Clostridia | Clostridiales | Lachnospiraceae |  |  | 131 |
| Firmicutes | Clostridia | Clostridiales |  |  |  | 132 |
| Firmicutes | Clostridia | Clostridiales | Lachnospiraceae |  |  | 133 |
| Firmicutes | Clostridia | Clostridiales | Lachnospiraceae |  |  | 134 |
| Firmicutes | Clostridia | Clostridiales | Lachnospiraceae |  |  | 135 |
| Firmicutes | Clostridia | Clostridiales | Ruminococcaceae | Oscillospira |  | 136 |
| Firmicutes | Clostridia | Clostridiales | Lachnospiraceae |  |  | 137 |
| Firmicutes | Clostridia | Clostridiales |  |  |  | 138 |
| Firmicutes | Clostridia | Clostridiales | Lachnospiraceae |  |  | 139 |
| Firmicutes | Clostridia | Clostridiales | Ruminococcaceae | Oscillospira |  | 141 |
| Firmicutes | Clostridia | Clostridiales |  |  |  | 142 |
| Firmicutes | Clostridia | Clostridiales | Ruminococcaceae |  |  | 143 |
| Firmicutes | Clostridia | Clostridiales |  |  |  | 144 |

| Firmicutes | Clostridia | Clostridiales | Ruminococcaceae | Ruminococcus |  | 145 |
| --- | --- | --- | --- | --- | --- | --- |
| Firmicutes | Clostridia | Clostridiales |  |  |  | 146 |
| Firmicutes | Clostridia | Clostridiales |  |  |  | 147 |
| Firmicutes | Bacilli | Lactobacillales | Enterococcaceae | Enterococcus |  | 149 |
| Firmicutes | Clostridia | Clostridiales | Lachnospiraceae |  |  | 150 |
| Firmicutes | Clostridia | Clostridiales |  |  |  | 152 |
| Firmicutes | Clostridia | Clostridiales |  |  |  | 153 |
| Firmicutes | Clostridia | Clostridiales | Ruminococcaceae | Ruminococcus |  | 155 |
| Firmicutes | Clostridia | Clostridiales | Ruminococcaceae |  |  | 156 |
| Firmicutes | Clostridia | Clostridiales | Ruminococcaceae | Oscillospira |  | 158 |
| Firmicutes | Clostridia | Clostridiales | Ruminococcaceae |  |  | 159 |
| Firmicutes | Bacilli | Lactobacillales | Streptococcaceae | Streptococcus |  | 160 |
| Firmicutes | Clostridia | Clostridiales | Ruminococcaceae |  |  | 161 |
| Firmicutes | Erysipelotrichi | Erysipelotrichales | Erysipelotrichaceae |  |  | 163 |
| Firmicutes | Clostridia | Clostridiales | Ruminococcaceae | Oscillospira |  | 165 |
| Firmicutes | Clostridia | Clostridiales | Ruminococcaceae | Oscillospira |  | 166 |
| Firmicutes | Clostridia | Clostridiales | Ruminococcaceae | Oscillospira |  | 167 |
| Firmicutes | Clostridia | Clostridiales | Ruminococcaceae |  |  | 168 |
| Firmicutes | Clostridia | Clostridiales | Lachnospiraceae | Dorea |  | 169 |
| Firmicutes | Clostridia | Clostridiales |  |  |  | 170 |
| Firmicutes | Clostridia | Clostridiales |  |  |  | 171 |
| Firmicutes | Clostridia | Clostridiales |  |  |  | 172 |
| Firmicutes | Clostridia | Clostridiales |  |  |  | 174 |
| Firmicutes | Clostridia | Clostridiales | Dehalobacteriaceae | Dehalobacterium |  | 176 |
| Firmicutes | Erysipelotrichi | Erysipelotrichales | Erysipelotrichaceae | Coprobacillus |  | 177 |
| Firmicutes | Clostridia | Clostridiales |  |  |  | 178 |
| Firmicutes | Clostridia | Clostridiales | Ruminococcaceae | Ruminococcus |  | 179 |
| Firmicutes | Clostridia | Clostridiales |  |  |  | 181 |
| Firmicutes | Clostridia | Clostridiales |  |  |  | 182 |
| Firmicutes | Clostridia | Clostridiales |  |  |  | 183 |
| Firmicutes | Clostridia | Clostridiales | Ruminococcaceae | Ruminococcus |  | 184 |
| Firmicutes | Clostridia | Clostridiales |  |  |  | 185 |
| Firmicutes | Clostridia | Clostridiales | [Mogibacteriaceae] |  |  | 186 |
| Firmicutes | Clostridia | Clostridiales | Dehalobacteriaceae | Dehalobacterium |  | 187 |
| Firmicutes | Clostridia | Clostridiales | Lachnospiraceae |  |  | 188 |
| Firmicutes | Clostridia | Clostridiales | Lachnospiraceae |  |  | 189 |
| Firmicutes | Clostridia | Clostridiales | Ruminococcaceae |  |  | 190 |
| Firmicutes | Clostridia | Clostridiales | Lachnospiraceae |  |  | 191 |
| Firmicutes | Clostridia | Clostridiales | Ruminococcaceae |  |  | 192 |
| Firmicutes | Clostridia | Clostridiales |  |  |  | 195 |
| Firmicutes | Clostridia | Clostridiales |  |  |  | 196 |
| Firmicutes | Clostridia | Clostridiales | Lachnospiraceae |  |  | 197 |
| Firmicutes | Clostridia | Clostridiales | Lachnospiraceae |  |  | 198 |

| Firmicutes | Clostridia | Clostridiales |  |  |  | 200 |
| --- | --- | --- | --- | --- | --- | --- |
| Firmicutes | Clostridia | Clostridiales | [Mogibacteriaceae] |  |  | 201 |
| Firmicutes | Clostridia | Clostridiales |  |  |  | 202 |
| Firmicutes | Clostridia | Clostridiales | Ruminococcaceae | Ruminococcus |  | 203 |
| Firmicutes | Clostridia | Clostridiales | Ruminococcaceae | Oscillospira |  | 204 |
| Firmicutes | Clostridia | Clostridiales |  |  |  | 205 |
| Firmicutes | Clostridia | Clostridiales | Ruminococcaceae | Oscillospira |  | 208 |
| Firmicutes | Clostridia | Clostridiales |  |  |  | 209 |
| Firmicutes | Clostridia | Clostridiales | Lachnospiraceae |  |  | 210 |
| Firmicutes | Erysipelotrichi | Erysipelotrichales | Erysipelotrichaceae |  |  | 212 |
| Firmicutes | Clostridia | Clostridiales | Lachnospiraceae |  |  | 214 |
| Firmicutes | Clostridia | Clostridiales |  |  |  | 215 |
| Firmicutes | Clostridia | Clostridiales |  |  |  | 216 |
| Firmicutes | Erysipelotrichi | Erysipelotrichales | Erysipelotrichaceae |  |  | 218 |
| Firmicutes | Clostridia | Clostridiales | Lachnospiraceae | Coprococcus |  | 219 |
| Firmicutes | Clostridia | Clostridiales | Ruminococcaceae | Ruminococcus |  | 220 |
| Firmicutes | Clostridia | Clostridiales | Ruminococcaceae |  |  | 221 |
| Firmicutes | Erysipelotrichi | Erysipelotrichales | Erysipelotrichaceae | Coprobacillus |  | 222 |
| Firmicutes | Clostridia | Clostridiales |  |  |  | 223 |
| Firmicutes | Clostridia | Clostridiales | Ruminococcaceae |  |  | 225 |
| Firmicutes | Clostridia | Clostridiales | Ruminococcaceae |  |  | 227 |
| Firmicutes | Clostridia | Clostridiales | Lachnospiraceae | Dorea |  | 230 |
| Firmicutes | Clostridia | Clostridiales | Peptococcaceae |  |  | 231 |
| Firmicutes | Erysipelotrichi | Erysipelotrichales | Erysipelotrichaceae |  |  | 232 |
| Firmicutes | Clostridia | Clostridiales |  |  |  | 233 |
| Firmicutes | Clostridia | Clostridiales |  |  |  | 234 |
| Firmicutes | Clostridia | Clostridiales |  |  |  | 235 |
| Firmicutes | Clostridia | Clostridiales | Ruminococcaceae | Oscillospira |  | 236 |
| Firmicutes | Clostridia | Clostridiales |  |  |  | 237 |
| Firmicutes | Clostridia | Clostridiales |  |  |  | 238 |
| Firmicutes | Clostridia | Clostridiales | [Mogibacteriaceae] |  |  | 239 |
| Firmicutes | Clostridia | Clostridiales |  |  |  | 240 |
| Firmicutes | Clostridia | Clostridiales | Lachnospiraceae |  |  | 241 |
| Firmicutes | Erysipelotrichi | Erysipelotrichales | Erysipelotrichaceae |  |  | 242 |
| Firmicutes | Clostridia | Clostridiales | Lachnospiraceae | Coprococcus |  | 244 |
| Firmicutes | Clostridia | Clostridiales | Ruminococcaceae |  |  | 247 |
| Firmicutes | Clostridia | Clostridiales |  |  |  | 248 |
| Firmicutes | Clostridia | Clostridiales | Lachnospiraceae |  |  | 249 |
| Firmicutes | Clostridia | Clostridiales |  |  |  | 253 |
| Firmicutes | Clostridia | Clostridiales |  |  |  | 254 |
| Firmicutes | Clostridia | Clostridiales |  |  |  | 257 |
| Firmicutes | Clostridia | Clostridiales | Lachnospiraceae |  |  | 258 |
| Firmicutes | Clostridia | Clostridiales | Ruminococcaceae | Ruminococcus |  | 259 |

| Firmicutes | Clostridia | Clostridiales | Lachnospiraceae |  |  | 260 |
| --- | --- | --- | --- | --- | --- | --- |
| Firmicutes | Clostridia | Clostridiales |  |  |  | 262 |
| Firmicutes | Clostridia | Clostridiales | Lachnospiraceae | Coprococcus |  | 264 |
| Firmicutes | Erysipelotrichi | Erysipelotrichales | Erysipelotrichaceae |  |  | 265 |
| Firmicutes | Clostridia | Clostridiales | Ruminococcaceae |  |  | 266 |
| Firmicutes | Clostridia | Clostridiales | Ruminococcaceae | Oscillospira |  | 267 |
| Firmicutes | Clostridia | Clostridiales | [Mogibacteriaceae] |  |  | 269 |
| Firmicutes | Clostridia | Clostridiales |  |  |  | 271 |
| Firmicutes | Clostridia | Clostridiales | Ruminococcaceae |  |  | 272 |
| Firmicutes | Clostridia | Clostridiales |  |  |  | 273 |
| Firmicutes | Clostridia | Clostridiales | Ruminococcaceae | Ruminococcus |  | 274 |
| Firmicutes | Clostridia | Clostridiales |  |  |  | 276 |
| Firmicutes | Clostridia | Clostridiales | Ruminococcaceae | Oscillospira |  | 277 |
| Firmicutes | Clostridia | Clostridiales |  |  |  | 278 |
| Firmicutes | Clostridia | Clostridiales | Christensenellaceae |  |  | 279 |
| Firmicutes | Clostridia | Clostridiales |  |  |  | 280 |
| Firmicutes | Clostridia | Clostridiales | Clostridiaceae | Clostridium |  | 281 |
| Firmicutes | Clostridia | Clostridiales |  |  |  | 283 |
| Firmicutes | Clostridia | Clostridiales | Ruminococcaceae |  |  | 284 |
| Firmicutes | Clostridia | Clostridiales |  |  |  | 286 |
| Firmicutes | Clostridia | Clostridiales |  |  |  | 287 |
| Firmicutes | Clostridia | Clostridiales |  |  |  | 289 |
| Firmicutes | Clostridia | Clostridiales |  |  |  | 290 |
| Firmicutes | Clostridia | Clostridiales | Clostridiaceae |  |  | 291 |
| Firmicutes | Clostridia | Clostridiales | Lachnospiraceae |  |  | 293 |
| Firmicutes | Clostridia | Clostridiales |  |  |  | 294 |
| Firmicutes | Clostridia | Clostridiales | Lachnospiraceae |  |  | 295 |
| Firmicutes | Clostridia | Clostridiales | Ruminococcaceae | Oscillospira |  | 296 |
| Firmicutes | Clostridia | Clostridiales | Ruminococcaceae | Oscillospira |  | 297 |
| Firmicutes | Clostridia | Clostridiales | Lachnospiraceae | Anaerostipes |  | 298 |
| Firmicutes | Clostridia | Clostridiales | Ruminococcaceae |  |  | 299 |
| Firmicutes | Clostridia | Clostridiales | Ruminococcaceae |  |  | 300 |
| Firmicutes | Clostridia | Clostridiales |  |  |  | 302 |
| Firmicutes | Erysipelotrichi | Erysipelotrichales | Erysipelotrichaceae |  |  | 303 |
| Firmicutes | Clostridia | Clostridiales | Ruminococcaceae | Ruminococcus |  | 304 |
| Firmicutes | Clostridia | Clostridiales | Ruminococcaceae |  |  | 305 |
| Firmicutes | Clostridia | Clostridiales | Ruminococcaceae | Ruminococcus |  | 308 |
| Firmicutes | Clostridia | Clostridiales | Ruminococcaceae |  |  | 309 |
| Firmicutes | Clostridia | Clostridiales |  |  |  | 311 |
| Firmicutes | Clostridia | Clostridiales |  |  |  | 312 |
| Firmicutes | Clostridia | Clostridiales |  |  |  | 313 |
| Firmicutes | Clostridia | Clostridiales | [Mogibacteriaceae] |  |  | 314 |
| Firmicutes | Erysipelotrichi | Erysipelotrichales | Erysipelotrichaceae |  |  | 315 |

| Firmicutes | Clostridia | Clostridiales | Lachnospiraceae |  |  | 316 |
| --- | --- | --- | --- | --- | --- | --- |
| Firmicutes | Clostridia | Clostridiales |  |  |  | 318 |
| Firmicutes | Clostridia | Clostridiales |  |  |  | 320 |
| Firmicutes | Clostridia | Clostridiales | Ruminococcaceae |  |  | 321 |
| Firmicutes | Clostridia | Clostridiales | Lachnospiraceae | Coprococcus |  | 322 |
| Firmicutes | Clostridia | Clostridiales | Ruminococcaceae |  |  | 323 |
| Firmicutes | Clostridia | Clostridiales | Lachnospiraceae | [Ruminococcus] | gnavus | 324 |
| Firmicutes | Clostridia | Clostridiales | Peptostreptococcaceae |  |  | 325 |
| Firmicutes | Clostridia | Clostridiales |  |  |  | 326 |
| Proteobacteria | Gammaproteobacteria | Enterobacteriales | Enterobacteriaceae |  |  | 2 |
| Proteobacteria | Deltaproteobacteria | Desulfovibrionales | Desulfovibrionaceae | Desulfovibrio |  | 44 |
| Proteobacteria | Deltaproteobacteria | Desulfovibrionales | Desulfovibrionaceae |  |  | 58 |
| Proteobacteria | Betaproteobacteria | Burkholderiales | Alcaligenaceae | Sutterella |  | 73 |
| Proteobacteria | Deltaproteobacteria | Desulfovibrionales | Desulfovibrionaceae | Desulfovibrio |  | 81 |
| Proteobacteria | Alphaproteobacteria |  |  |  |  | 213 |
| Tenericutes | Mollicutes | RF39 |  |  |  | 38 |
| Tenericutes | Mollicutes | Anaeroplasmatales | Anaeroplasmataceae | Anaeroplasma |  | 63 |
| Tenericutes | Mollicutes | RF39 |  |  |  | 162 |
| Tenericutes | Mollicutes | Anaeroplasmatales | Anaeroplasmataceae | Anaeroplasma |  | 180 |
| Tenericutes | Mollicutes | RF39 |  |  |  | 224 |
| TM7 | TM7-3 | CW040 | F16 |  |  | 23 |
| TM7 | TM7-3 | CW040 | F16 |  |  | 261 |
| Unassigned | Unassigned | Unassigned | Unassigned | Unassigned | Unassigned | 5 |
| Unassigned | Unassigned | Unassigned | Unassigned | Unassigned | Unassigned | 45 |
| Unassigned | Unassigned | Unassigned | Unassigned | Unassigned | Unassigned | 245 |
| Unassigned | Unassigned | Unassigned | Unassigned | Unassigned | Unassigned | 246 |
| Unassigned | Unassigned | Unassigned | Unassigned | Unassigned | Unassigned | 255 |
| Unassigned | Unassigned | Unassigned | Unassigned | Unassigned | Unassigned | 282 |
| Unassigned | Unassigned | Unassigned | Unassigned | Unassigned | Unassigned | 288 |
| Unassigned | Unassigned | Unassigned | Unassigned | Unassigned | Unassigned | 306 |
| Unassigned | Unassigned | Unassigned | Unassigned | Unassigned | Unassigned | 319 |

**Supplementary Table 2. Spearman correlations**

Correlation coefficients and p-values calculated from spearman correlation. All P-values were corrected for multiple testing with FDR, correlations for cecum content samples are in table 6-8

and tissue samples are in table 9-11.

(A) OUTs with significant correlation to age in cecum content, p-value and rank coefficient.

| **OTU number** | **p-value** | **Coeff** |
| --- | --- | --- |
| 9 | 0,0000 | 0,7651 |
| 13 | 0,0113 | 0,4400 |
| 29 | 0,0085 | 0,4551 |
| 30 | 0,0143 | 0,4238 |
| 32 | 0,0174 | 0,4134 |
| 36 | 0,0028 | -0,4989 |
| 41 | 0,0001 | 0,6038 |
| 47 | 0,0000 | 0,6279 |
| 48 | 0,0393 | -0,3746 |
| 49 | 0,0141 | 0,4258 |
| 51 | 0,0002 | 0,5809 |
| 62 | 0,0002 | -0,5691 |
| 72 | 0,0083 | -0,4595 |
| 73 | 0,0137 | 0,4291 |
| 84 | 0,0007 | 0,5420 |
| 85 | 0,0054 | 0,4763 |
| 89 | 0,0318 | -0,3859 |
| 90 | 0,0097 | 0,4491 |
| 108 | 0,0002 | -0,5736 |
| 120 | 0,0084 | 0,4570 |
| 121 | 0,0141 | 0,4255 |
| 126 | 0,0016 | 0,5181 |
| 127 | 0,0113 | 0,4397 |
| 130 | 0,0434 | 0,3697 |
| 131 | 0,0028 | -0,5014 |
| 135 | 0,0116 | -0,4374 |
| 142 | 0,0000 | -0,6319 |
| 143 | 0,0274 | 0,3937 |
| 148 | 0,0362 | 0,3789 |
| 154 | 0,0137 | 0,4306 |
| 157 | 0,0055 | 0,4736 |
| 170 | 0,0110 | 0,4435 |
| 179 | 0,0041 | 0,4859 |
| 184 | 0,0154 | 0,4192 |
| 185 | 0,0274 | 0,3940 |

(B) OUTs with significant correlation to tumorload in the small intestine, p-value and rank coefficient.

| **OTU number** | **P-value** | **Coeff.** |
| --- | --- | --- |
| 9 | 0,0000 | 0,6738 |
| 13 | 0,0057 | 0,4873 |
| 29 | 0,0364 | 0,3977 |
| 36 | 0,0149 | -0,4477 |
| 41 | 0,0092 | 0,4675 |
| 47 | 0,0013 | 0,5282 |
| 48 | 0,0113 | -0,4586 |
| 49 | 0,0278 | 0,4161 |
| 51 | 0,0002 | 0,5783 |
| 62 | 0,0005 | -0,5563 |
| 72 | 0,0167 | -0,4394 |
| 73 | 0,0362 | 0,4004 |
| 84 | 0,0190 | 0,4330 |
| 108 | 0,0008 | -0,5450 |
| 125 | 0,0340 | 0,4053 |
| 126 | 0,0000 | 0,6311 |
| 130 | 0,0260 | 0,4204 |
| 131 | 0,0092 | -0,4691 |
| 139 | 0,0482 | -0,3840 |
| 142 | 0,0000 | -0,6296 |
| 157 | 0,0362 | 0,3996 |
| 216 | 0,0442 | 0,3889 |
| 254 | 0,0331 | 0,4081 |
| 257 | 0,0001 | -0,6069 |
| 271 | 0,0167 | 0,4413 |

| 190 | 0,0242 | 0,4006 |
| --- | --- | --- |
| 216 | 0,0154 | 0,4188 |
| 220 | 0,0318 | 0,3863 |
| 257 | 0,0001 | -0,6083 |
| 271 | 0,0362 | 0,3790 |

(C) OUTs with significant correlation to tumorload colon, p-value and rank coefficient.

| 127 | 0,0009 | 0,5151 |
| --- | --- | --- |
| 130 | 0,0001 | 0,5782 |
| 131 | 0,0000 | -0,6168 |
| 132 | 0,0271 | 0,3781 |
| 141 | 0,0237 | 0,3862 |
| 142 | 0,0000 | -0,6307 |
| 143 | 0,0008 | 0,5188 |
| 146 | 0,0311 | 0,3681 |
| 148 | 0,0093 | 0,4263 |
| 154 | 0,0005 | 0,5331 |
| 156 | 0,0378 | 0,3574 |
| 157 | 0,0000 | 0,6172 |
| 170 | 0,0250 | 0,3833 |
| 172 | 0,0279 | 0,3746 |
| 179 | 0,0052 | 0,4497 |
| 184 | 0,0001 | 0,5743 |
| 191 | 0,0428 | 0,3500 |
| 194 | 0,0023 | 0,4775 |
| 195 | 0,0271 | 0,3779 |
| 211 | 0,0144 | 0,4079 |
| 216 | 0,0011 | 0,5050 |
| 220 | 0,0164 | 0,4024 |
| 223 | 0,0279 | -0,3748 |
| 225 | 0,0036 | 0,4637 |
| 226 | 0,0020 | 0,4823 |
| 230 | 0,0354 | 0,3608 |
| 252 | 0,0308 | -0,3691 |
| 254 | 0,0100 | 0,4230 |
| 257 | 0,0000 | -0,6019 |
| 262 | 0,0011 | 0,5020 |
| 267 | 0,0186 | 0,3970 |
| 271 | 0,0428 | 0,3504 |
| 292 | 0,0429 | 0,3488 |
| 318 | 0,0455 | 0,3449 |
| 324 | 0,0016 | 0,4907 |

| **OTU number** | **P-value** | **Coeff** |
| --- | --- | --- |
| 9 | 0,0000 | 0,7069 |
| 13 | 0,0001 | 0,5909 |
| 15 | 0,0347 | -0,3623 |
| 21 | 0,0058 | 0,4452 |
| 29 | 0,0000 | 0,6059 |
| 30 | 0,0078 | 0,4328 |
| 32 | 0,0115 | 0,4173 |
| 36 | 0,0458 | -0,3440 |
| 39 | 0,0394 | -0,3549 |
| 41 | 0,0005 | 0,5307 |
| 47 | 0,0001 | 0,5702 |
| 48 | 0,0042 | -0,4569 |
| 49 | 0,0001 | 0,5806 |
| 51 | 0,0003 | 0,5465 |
| 56 | 0,0069 | 0,4377 |
| 59 | 0,0455 | -0,3450 |
| 62 | 0,0001 | -0,5706 |
| 72 | 0,0037 | -0,4619 |
| 73 | 0,0010 | 0,5095 |
| 79 | 0,0428 | 0,3495 |
| 84 | 0,0005 | 0,5323 |
| 88 | 0,0012 | 0,5006 |
| 89 | 0,0133 | -0,4114 |
| 90 | 0,0005 | 0,5364 |
| 98 | 0,0322 | 0,3660 |
| 101 | 0,0291 | -0,3722 |
| 105 | 0,0279 | 0,3753 |
| 107 | 0,0011 | 0,5049 |
| 108 | 0,0012 | -0,4993 |
| 113 | 0,0069 | 0,4386 |
| 116 | 0,0269 | 0,3797 |
| 118 | 0,0233 | -0,3876 |
| 120 | 0,0009 | 0,5125 |
| 121 | 0,0000 | 0,6234 |
| 126 | 0,0000 | 0,6830 |

(D) OUTs with significant correlation to age in tissue samples, p-value and rank coefficient.

| **OTU number** | **P-value** | **Coeff** |
| --- | --- | --- |
| 13 | 0,0246 | 0,5185 |
| 48 | 0,0444 | -0,4916 |
| 62 | 0,0048 | -0,5851 |
| 94 | 0,0166 | -0,5394 |
| 108 | 0,0000 | -0,7079 |

(F) OUTs with significant correlation to tumor progression in colon in tissue samples, p-value and rank coefficient.

(E) OUTs with significant correlation to tumor progression in the small intestine in tissue samples, p-value and rank coefficient.

| **OTU number** | **P-value** | **Coeff** |
| --- | --- | --- |
| 7 | 0,0355 | 0,4324 |
| 9 | 0,0465 | 0,4121 |
| 13 | 0,0047 | 0,5854 |
| 29 | 0,0113 | 0,5144 |
| 32 | 0,0317 | 0,4715 |
| 39 | 0,0050 | -0,5731 |
| 41 | 0,0321 | 0,4611 |
| 47 | 0,0453 | 0,4157 |
| 49 | 0,0317 | 0,4695 |
| 58 | 0,0321 | 0,4508 |
| 59 | 0,0376 | -0,4261 |
| 62 | 0,0321 | -0,4609 |
| 84 | 0,0321 | 0,4520 |
| 86 | 0,0321 | -0,4632 |
| 97 | 0,0355 | 0,4334 |
| 105 | 0,0355 | 0,4413 |
| 108 | 0,0057 | -0,5621 |
| 110 | 0,0113 | 0,5214 |
| 113 | 0,0395 | 0,4227 |
| 120 | 0,0460 | 0,4138 |
| 126 | 0,0321 | 0,4548 |
| 127 | 0,0113 | 0,5141 |
| 130 | 0,0355 | 0,4384 |
| 133 | 0,0321 | 0,4490 |
| 142 | 0,0112 | -0,5305 |
| 147 | 0,0139 | 0,5042 |
| 148 | 0,0093 | 0,5415 |
| 157 | 0,0047 | 0,5931 |
| 170 | 0,0355 | 0,4362 |
| 171 | 0,0364 | -0,4287 |
| 172 | 0,0355 | 0,4346 |
| 179 | 0,0364 | 0,4294 |
| 216 | 0,0355 | 0,4342 |
| 226 | 0,0321 | 0,4559 |
| 262 | 0,0113 | 0,5184 |
| 298 | 0,0321 | 0,4541 |
| 306 | 0,0355 | -0,4425 |

| **OTU number** | **P-value** | **Coeff** |
| --- | --- | --- |
| 9 | 0,0176 | 0,5112 |
| 13 | 0,0092 | 0,5484 |
| 36 | 0,0310 | -0,4676 |
| 41 | 0,0092 | 0,5520 |
| 51 | 0,0222 | 0,4951 |
| 52 | 0,0152 | 0,5266 |
| 62 | 0,0024 | -0,6190 |
| 84 | 0,0176 | 0,5133 |
| 108 | 0,0070 | -0,5751 |
| 171 | 0,0238 | -0,4859 |
| 179 | 0,0275 | 0,4746 |
| 185 | 0,0222 | 0,4953 |
| 190 | 0,0483 | 0,4430 |
| 198 | 0,0316 | 0,4643 |
| 237 | 0,0483 | 0,4435 |
| 262 | 0,0234 | 0,4898 |
| 306 | 0,0264 | -0,4790 |
